# Health status and self‐management in patients with inflammatory arthritis—A five‐year follow‐up study after nurse‐led patient education

**DOI:** 10.1002/nop2.394

**Published:** 2019-10-08

**Authors:** Kjersti Grønning, Siriwan Lim, Ola Bratås

**Affiliations:** ^1^ Department of Public Health and Nursing Norwegian University of Science and Technology (NTNU) Trondheim Norway; ^2^ Department of Rheumatology Trondheim University Hospital St.Olavs Hospital Prinsesse Kristinas gate 3 Trondheim Norway; ^3^ Alice Lee Centre for Nursing Studies Yong Loo Lin School of Medicine National University of Singapore Singapore Singapore

**Keywords:** chronic inflammatory arthritis, health status, nurse‐led care, patient education, self‐efficacy, self‐management

## Abstract

**Aim:**

The aim of this study was to investigate changes in patients' self‐management and health status five years after nurse‐led patient education.

**Design:**

A longitudinal study.

**Methods:**

We collected self‐reported data on physical function, pain, tiredness, disease activity, psychological status, patient activation and self‐efficacy from a sample of Norwegian‐speaking adults with inflammatory arthritis that had participated in a randomised controlled study investigating the effects of nurse‐led patient education. Changes and associations in patients' health status and self‐management were analysed with paired sample *t* tests and multivariable linear regression analyses, respectively.

**Results:**

Except from a small deterioration in patients' physical function, there were no changes in patients' health status 5 years after the nurse‐led patient education. Patients' self‐management skills were improved after 5 years. Self‐efficacy was positively associated with female gender, patient activation, less tiredness and less psychological distress.

## 1 INTRODUCTION

Chronic inflammatory arthritis (IA) including rheumatoid arthritis (RA), psoriatic arthritis (PsA) or unspecified polyarthritis (UA) are all chronic diseases with various degree of symptoms like joint inflammation, joint tenderness, stiffness, pain and fatigue (Ledingham, Snowden, & Ide, 2017). Patient education is recommended as an integral part of disease management for people with IA (Combe et al., 2017; Deighton, O'Mahony, Tosh, Turner, & Rudolf, 2009; Dejaco et al., 2015; Richette et al., 2017; Smolen et al., 2016; van der Heijde et al., 2017; Zangi et al., 2015), where long‐term pharmacological treatment (Connelly et al., 2018) and disease activity self‐assessment are central. In addition to patient education, patients need support after attendance, often provided as follow‐up phone calls or face‐to‐face consultations (Vivienne & Michael, 2018).

## 2 BACKGROUND

The treatment of IA has improved dramatically in later years due to the introduction of biological disease‐modifying anti‐rheumatic drugs (bDMARDs) and early onset of effective treatment (“treat‐to‐target” principle) (Smolen, 2016). Early diagnosis and treatment are crucial to improve function, radiographic outcomes and prevent work disability (Olofsson et al., 2017). Supporting people to stay employed is important and associated with increased quality of life (Grønning, Rødevand, & Steinsbekk, 2010) and emphasized as important by patients (Grønning, Lomundal, Koksvik, & Steinsbekk, 2011). Although patients with IA experience that coping is challenging, they make daily adjustments to handle the disease fluctuations to live as normal and good lives as possible (Grønning et al., 2011). They also perceive that support from others with similar experiences is uniquely valuable (Hughes et al., 2017). However, patients with IA have a higher predisposition to develop depression or psychological distress (Howells et al., 2017; Vallerand, Patten, & Barnabe, 2019). Depression increases the risk of disease flares; decrease rates of remission and interferes with patients' coping strategies, clinical management (e.g. medication compliance) and quality of life (Vallerand et al., 2019). In the opposite case, patients with less depressed mood and dissatisfaction with life have higher levels of self‐efficacy and role balance (Coty, Salt, Myers, & Abusalem, 2017). However, lifestyle habits and choices (Bandura, 2004) do also influence patients' health and well‐being where good self‐management is essential to live good lives. Albert Bandura (Bandura, 2004) states that knowledge is important for people's motivation to make changes or adjustments in life. If people do not have enough knowledge about how their life or coping styles affect their health, they have fewer reasons to make changes to gain better health. The overarching principle in the field of rheumatology is that patient education should enable people to manage their life with IA and optimize their health and well‐being rather than be limited to the disease (Zangi et al., 2015). This overarching principle is similar for other chronic diseases as well (Prothero, Barley, Galloway, Georgopoulou, & Sturt, 2018; Stenberg, Haaland‐Overby, Haaland‐Øverby, Fredriksen, Westermann, & Kvisvik, 2016).

Several RCT studies on patient education for people with IA have shown positive effects on different outcomes such as self‐efficacy (Grønning et. al 2012, Zhao & Chen, 2019), pain, fatigue, illness perception, quality of life, sedentary time (Knittle et al.,^2015^) and overall well‐being (Grønning et. al 2012, Grønning et. al 2013). However, there is a need for longitudinal studies examining whether improvements in psychological status produce carry‐over effects on physical outcomes and which strategies that enhance patients' long‐term adherence to such programmes (Prothero et al., 2018). Therefore, the aim of this study was to investigate changes in self‐management and health status among patients with IA that had participated in an RCT on nurse‐led patient education 5 years earlier; secondly, to study associations between disease‐related factors, health status, demographics and self‐efficacy in the same sample of patients with IA.

## 3 DESIGN

This is a longitudinal five‐year follow‐up study of a sample of patients that participated in an RCT of hospital‐based nurse‐led patient education (Grønning, Rannestad, Skomsvoll, Rygg, & Steinsbekk, 2013; Grønning, Skomsvoll, Rannestad, & Steinsbekk, 2012) from 2008–2010 in mid‐Norway. The RCT was registered in Clinical Trials, NCT00623922. Eligible participants were identified by a research nurse who searched the hospital's electronic patient record system at the Department of Rheumatology, St. Olavs University Hospital. The participants received a written invitation letter about the purpose of the study. Then, they contacted the researcher to make a screening appointment. If the participants fulfilled the inclusion criteria, they were randomised to the intervention (nurse‐led patient education programme) or to the control group (usual care) after the baseline data collection. The randomisation was done by a computer‐based Internet trial service provided by the Norwegian University of Science and Technology. The intervention consisted of a combination of three group‐sessions and one individual educational session led by two nurses. It included lectures and group discussions about pathology, common symptoms, prognosis, symptom circle, coping skills, self‐management, motivation, goals, medical treatment, how to observe side effects, community resources, healthy lifestyle, exercise and diet. Usual care was regular follow‐up appointments at the Rheumatology department and/or general practitioners (GP) (Grønning et al., 2013, 2012). After the RCT was finished, all participants in the control group were offered to take part in the nurse‐led patient education.

## 4 METHOD

The inclusion criteria for the RCT were Norwegian‐speaking adults between 18 and 80 years old, having an IA diagnose (RA, PsA or UA), while having participated in patient education one year prior to the RCT was an exclusion criterium (Grønning et al., 2013, 2012). Data were collected at baseline in the RCT (T0 = 2008–2009), 12 months later (final follow‐up in the RCT) and 5 years later (T2 = 2015–2016). The data consisted of demographic information, disease characteristics and domains that patient education may influence (Prothero et al., 2018) such as physical function, disease symptoms (pain, tiredness and disease activity), psychological status (well‐being, anxiety and depression) and self‐management (patient activation and self‐efficacy). An objective measure of disease activity, the disease activity score (DAS28‐3) using 28 tender and swollen joints counts (van Riel & Renskers, 2017), was only collected in the RCT and not in the five‐year follow‐up study. All questionnaires were translated into Norwegian and found valid for patients with IA. They consisted of the modified health assessment questionnaires (MHAQ) (Pincus, Summey, Soraci, Wallston, & Hummon, 1983) which evaluate difficulties in getting dressed, getting up, mobility, hygienic, grip and activity in patients with IA (Kvien, Kaasa, & Smedstad, 1998). Patients' experiences of pain, tiredness and disease activity were measured using three 100 mm visual analogue scales (VASs) (Lati, Guthrie, & Ward, 2010). Patients' overall well‐being (last month) was assessed by the Arizona Integrative Outcomes Scale (AIOS) (Bell, Cunningham, Caspi, Meek, & Ferro, 2004), the Hospital Anxiety and Depression Scale (HADS) (Bjelland, Dahl, Haug, & Neckelmann, 2002; Zigmond & Snaith, 1983) was used to measure psychological status. Self‐efficacy was measured using two sub‐scales: the arthritis self‐efficacy other symptoms (SE symptoms) and pain (SE pain) scales (Lorig, Chastain, Ung, Shoor, & Holman, 1989, (Brekke, Hjortdahl, & Kvien, 2003) while self‐management was measured by the patient activation measure (PAM‐13) (Hibbard, Mahoney, Stockard, & Tusler, 2005, Steinsbekk, 2008). SE symptoms and SE pain capture patients' beliefs in their abilities to cope with arthritis symptoms and pain while PAM‐13 captures patients' knowledge, skills, beliefs and behaviours in managing chronic illness.

### 4.1 Analyses

Paired *t* tests were used to analyse changes from between the final follow‐up in the RCT and 5 years later (Altman, 1991). Associations between the dependent variables (SE other symptoms and SE pain) and the independent variables (age, gender, disease characteristics, MHAQ, VAS pain, tiredness, disease activity, AIOS, HADS and PAM‐13) were analysed with multivariable linear regression analyses. These variables were chosen as independent variables because there is a need to better understand the pathways of the relationships between self‐efficacy and psychological distress (Benka et al., 2014) and if physical disease‐related variables affect self‐efficacy (Primdahl, Wagner, & Horslev‐Petersen, 2011). In multivariable regression analyses, the standardized beta coefficient (Beta) compares the strength of the association between the independent and dependent variable when controlling for other independent variables in the model. The level of significance was set to *p* < .05. The assumptions of linear regression analyses were checked, finding the Durbin–Watson and variance inflation factor both satisfactory. Contribution of the independent variables in the model is expressed as explained variance (adjusted *R* ^2^). Missing data were deleted listwise as our data were missing completely at random. The data were analysed using IBM SPSS Statistics (version 24) (SPSS, 2016).

### 4.2 Ethical considerations

The regional committee for medical and health research ethics in South East Norway (2014/196/REK sør‐øst A) approved the study. The participants signed a written consent to participate in the study and returned their written consent in the same pre‐paid envelope as the self‐reported questionnaires.

## 5 RESULTS

The sample in this study consisted of 101 patients. There were 132 eligible patients at the final follow‐up in the completed RCT (Grønning et al., 2013). Five patients had passed away when the invitation letter to participate in this study was sent to eligible patients (*N* = 127). We received 101 envelopes containing signed consents to participate and completed questionnaires, four envelopes containing no data and two envelopes were returned and marked with “unknown addresses.”

The sample's characteristics are presented in Table 1, showing that the participants consisted of more women than men, most were living with someone (married or co‐habiting), most patients were diagnosed with RA, approximately half of the sample reported to have one or several additional diseases and their disease activity score was moderate.

**Table 1:** Participant characteristic at baseline (*N* = 101)

	*N*	%	Mean	*SD*
Men	29	28.7		
Females	72	71.3		
Co‐habiting	86	85.1		
Age			58.7	9.9
Education (university level or more)	34	33.7		
RA	63	62.4		
PsA	20	19.8		
UA	15	14.9		
Disease duration			11.5	9.3
Comorbidities	56	55.4		
Using DMARDs	82	83		
DAS28−3			3.1	1.0

Abbreviations: DAS28‐3, disease activity score; DMARDS, disease‐modifying anti‐rheumatic drugs; PsA, psoriatic arthritis; RA, rheumatoid arthritis; UA, unspecified polyarthritis.

In the intervention group, 47 patients attended the nurse‐led patient education programme. In the control group, 37 patients reported that they wanted to attend the patient education programme after the RCT was completed, while 7 patients said they did not need any education after all, and 4 patients said they would contact the department later on if they felt in need of patient education.

### 5.1 Changes in health status and self‐management

Changes in patients' self‐management and health status are presented in Table 2, showing that patients' health status was stable from T1 to T2 except from a small worsening in physical function (MHAQ mean change score of 0.12, CI 0.01–0.24, *p*‐value .035). The participants' self‐management as assessed by PAM‐13 was improved, (PAM‐13 mean change score of 3.69, CI 0.50–6.88, *p*‐value .024), but there were no statistically significant changes in patients' self‐efficacy. There were no changes in patients' well‐being, psychological status, perceived pain, tiredness or self‐reported disease activity.

**Table 2:** Changes in health status, disease symptoms and self‐management (*n* = 101)

	T1	T2	Within changes	*p*‐value
	Mean (*SD*)		Mean difference (95% CI)	
AIOS (0–100) ↑	55.3 (22.4)	53.7 (18.2)	−1.62 (−7.05 to 3.8)	.554
SE symptoms (10–100) ↑	67.9 (16.9)	68.8 (16.8)	0.82 (−2.41 to 4.06)	.615
SE pain (10–100) ↑	59.2 (19.6)	61.7 (19.5)	2.49 (−1.22 to 6.20)	.186
PAM 13 (0–100) ↑	66.1 (16.9)	69.8 (14.5)	3.69 (0.50 to 6.88)	.024*
MHAQ (0–3) ↓	1.5 (0.4)	1.6 (0.6)	0.12 (0.01 to 0.24)	.035*
HADS total (0–42) ↓	9.4 (6.5)	9.8 (7.3)	0.40 (−0.62 to 1.41)	.442
VAS pain (0–100) ↓	42.6 (23.6)	44.7 (22.9)	2.04 (−3.55 to 7.63)	.471
VAS tiredness (0–100) ↓	49.1 (26.1)	47.8 (24.1)	−1.29 (−6.77 to 4.19)	.642
VAS disease activity (0–100) ↓	43.0 (26.2)	44.7 (22.5)	1.69 (−4.55 to 7.93)	.592

Note

T1, Last follow‐up in the RCT, T2, 5 years later. Paired T tests, Level of significance.

Abbreviations: ↑, higher score is better; ↓, lower score is better; AIOS, Arizona Integrative Outcomes Scale; HADS, Hospital Anxiety and Depression Scale; MHAQ, modified health assessment questionnaire; PAM13, patient activation measure‐13; SE pain, self‐efficacy pain; SE symptoms, self‐efficacy other symptoms; VAS, 100 mm visual analogue scales.

* ***p* < .05** *.*

### 5.2 Self‐efficacy associations

The results from the multivariable regression analyses are presented in Table 3, showing that female gender, patient activation, psychological distress and tiredness had statistically significant independent effects on self‐efficacy other symptoms when controlling for education, age, well‐being, self‐management, physical function, pain and perceived disease activity. Female gender, higher scores of patient activation, less psychological distress and less tiredness were associated with better self‐efficacy in managing arthritis symptoms. Higher scores of patient activation and female gender were also associated with better self‐efficacy in managing pain when controlling for education, age, well‐being, psychological distress, tiredness, physical function, pain and perceived disease activity. The total variance in the model was 93 dfs while the residual dfs was 83. The regression models accounted for 52.6% and 35.0% of the explained variance (*R* ^2^) in SE symptoms and SE pain, respectively.

**Table 3:** Explained variance in self‐efficacy at five‐year follow‐up

Co‐ variates	Self‐efficacy other symptoms				Self‐efficacy pain			
	B	Std. Error	Beta	*p*‐value	B	Std. Error	Beta	*p*‐value
*Constant*	38.826	14.647		.010	48.716	19.901		.016
Female	7.074	2.798	0.194	.013*	12.589	3.801	0.298	.001*
University education	2.326	2.701	0.065	.392	3.944	3.670	0.096	.286
Age	0.052	0.133	0.031	.696	−0.258	0.181	−0.131	.158
AIOS (0–100) ↑	0.052	0.084	0.056	.540	0.011	0.114	0.011	.921
PAM 13 (0–100) ↑	0.446	0.107	0.380	<.001*	0.468	0.146	0.344	.002*
MHAQ (0–3) ↓	1.224	2.253	0.047	.588	0.704	3.061	0.023	.819
HADS total (0–42) ↓	−0.710	0.219	−0.307	.002*	0.086	0.298	0.032	.774
VAS pain (0–100) ↓	0.124	0.092	0.170	.184	−0.035	0.126	−0.041	.783
VAS tiredness (0–100) ↓	−0.146	0.057	−0.211	.012*	−0.155	0.077	−0.193	.048*
VAS disease activity (0–100) ↓	−0.131	0.096	−0.178	.175	−0.172	0.130	−0.201	.190
Adjusted R^2^				52.6				35.0

Note

B = Multiple linear regression analyses, unstandardized coefficients, Std. Error = standard error, Beta = standardized coefficients.

Abbreviations: ↑, higher score is better; ↓, lower score is better; AIOS, Arizona Integrative Outcomes Scale; HADS, Hospital Anxiety and Depression Scale; MHAQ, modified health assessment questionnaire; PAM13, patient activation measure‐13; VAS, 100 mm visual analogue scales.

* *p*‐value < .05.

## 6 DISCUSSION

One of the aims in this study was to investigate long‐term changes in patients' self‐management and health status 5 years after the patients had participated in a completed RCT on nurse‐led patient education. The results showed that patients' self‐management had increased during this period while patients' physical function had slightly deteriorated.

### 6.1 Self‐management support

In the field of rheumatology, nurses have an important role in the management of chronic inflammatory arthritis (Vivienne & Michael, 2018) together with medical doctors and other healthcare professionals. The main treatment goal is to reduce disease activity, prevent structural damage and improve functionality and social participation (Smolen, 2016). Our results, showing that the participants' health status (perceived pain, tiredness and disease activity) were unchanged, except from a small deterioration in physical function, indicate that the treatment goals probably were almost reached.

Also, an essential component in the care of patients with IA is to support patient's self‐management (Morgan et al., 2017) as patients need to learn how to balance their activities and energy according to their individual situations (Grønning et al., 2011). Supporting patient's self‐management includes focusing on self‐care, self‐efficacy, (Knittle et al., 2015) and empowerment (Arvidsson, Bergman, Arvidsson, Fridlund, & Tingstrom, 2013). In this study, the participants' level of self‐management measured by PAM‐13 had increased during the five‐year follow‐up period, indicating that the participants possessed a high degree of knowledge, skills and confidence to manage their disease. A patient activation level of 69.8 at the five‐year follow‐up (Table 2) demonstrates that the participants in this study had adopted and maintained favourable behaviours and self‐management strategies (Hibbard et al., 2005) and are active managers of their health and health care.

The second aim in this study was to study associations between disease‐related factors, health status, demographics and self‐efficacy in patients with IA. The analyses showed that level of patient activation and female gender predicted higher self‐efficacy in managing pain, while less psychological distress and tiredness in addition to female gender and patient activation level, predicted higher self‐efficacy in managing arthritis symptoms. Patients that can monitor their disease activity (Cheung et al., 2010) and take responsibility in managing their disease have high self‐management skills. The results from the multivariable regression analyses showed that having a high level of patient activation, which indicate that a person have good knowledge, skills and behaviours to manage the disease, was independently associated with higher self‐efficacy in coping with arthritis symptoms and pain. This is good news as self‐management is shown to improve health outcomes (Stenberg et al., 2016) and very important to address in the treatment and care of patients with several chronic diseases (Ree, Wiig, Manser, & Storm, 2019; Turner et al., 2018). Good self‐management is also shown to limit patients' fears related to the being chronic ill (Palominos et al., 2018) and a theme often addressed in patient education and self‐management programmes (Hardware, Johnson, Hale, Ndosi, & Adebajo, 2015; Stenberg et al., 2016).

Furthermore, the results from this study support the findings from Coty and colleagues (Coty et al., 2017) showing that psychological or mental well‐being influence patients' self‐efficacy and self‐management (Coty et al., 2017). In our study, enhanced self‐efficacy was associated with female gender. This finding indicates that men and women with arthritis manage and experience their diseases differently, which is also emphasized in several studies (Flurey, Hewlett, & Rodham, 2017; Flurey et al., 2016a, 2016b, 2016c; Gruszczynska & Knoll, 2015). One study (Flurey et al., 2016a) found that men employed fewer and different strategies than women to manage their condition, females have more positive social support, while men are concerned about retaining their masculine identity. Another study (Gruszczynska & Knoll, 2015) showed that men needed more time to adjust and adapt to their disease, while women reported more negative mood or distress than men. Together with the results from this study, showing that men with IA have lower self‐efficacy than women, confirm that self‐efficacy is an important factor to address in clinical nursing practice. Other researchers (Dures et al., 2016; Zuidema, Repping‐Wuts, Evers, Van Gaal, & Van Achterberg, 2015)have also stated that that individualized and tailored psychological support is needed throughout the disease trajectory, where nurses and other healthcare professionals must acknowledge that patients have different informational, emotional, social and practical needs (Zuidema et al., 2015). Also, to maintain or strengthen patients' health and quality of life, nurses need to see patients as equal partners that possess an expertise in living with their chronic illnesses (Ekman et al., 2011; McCormack et al., 2015; Miles, 2012).

### 6.2 Strengths and limitations

The strength of this study is the longitudinal design with a follow‐up period of 5 years, making it possible to monitor changes over time. However, the study has also some limitations and risks of bias. First, the sample was originally recruited for an RCT studying the effect of nurse‐led patient education (selection bias), which may limit the external validity of the results. However, when comparing the health status in our sample with other populations of patients with IA in Norway (Uhlig, Heiberg, Mowinckel, & Kvien, 2008) and abroad (Hammond, Bryan, & Hardy, 2008), it shows that this sample is similar to those populations and thus strengthen the external validity of the results. The risk of performance bias is considered small, since the control group were offered to take part in the nurse‐led patient education programme after the final follow‐up (12 months) in the RCT. Another possible limitation is the use of several outcome measures followed by a chance for multiple statistical testing. However, multiple statistical testing was not a problem in this study. We did not investigate long‐term effects of the nurse‐led patient education, we explored associations while controlling for possible confounders.

### 6.3 Clinical implications

According to the 2018 update of the EULAR recommendations for the role of the nurses in the management of chronic IA (Bech et al., 2019), patients shall have access to a nurse for education to improve knowledge of IA and its management throughout the course of their disease. The results from this study, showing that patients' health status was stable over time, are valuable to discuss with patients in nurse consultations or in patient education, since many patients have fears related to the being chronically ill (Palominos et al., 2018). Also, knowing that higher self‐efficacy scores are associated with lesser psychological distress and tiredness, underline that talking to patients about how to balance their activities and energy according to their individual situations (Grønning et al., 2011) is needed to support patients to become good self‐managers, (Bech et. al, 2019, Chaleshgar‐Kordasiabi, Enjezab, Akhlaghi, & Sabzmakan, 2018; Dures et al., 2016; Ostlund, Björk, Thyberg, Valtersson, & Sverker, 2018) and that men and women may have different needs (Flurey et al., 2017, 2016a, 2016b, 2016c; Gruszczynska & Knoll, 2015).

## 7 CONCLUSION

This study found that patients' self‐management skills had improved five years after the completed RCT on nurse‐led patient education and that patients' health status was unchanged, except from a slightly deterioration in physical function. Furthermore, the analyses showed that level of patient activation and female gender predicted higher self‐efficacy in managing pain, while less psychological distress and tiredness in addition to female gender and patient activation level predicted higher self‐efficacy in managing arthritis symptoms while controlling for age, education, physical function and perceived pain, disease activity and overall well‐being.

## CONFLICT OF INTERESTS

The authors declare no competing interests.

## AUTHOR CONTRIBUTIONS

KG: study plan, patient recruitment, data gathering, statistical analysis and manuscript drafting. SL and OB: interpretation of data analysis, and critical revision of manuscript. All authors: approval of the final manuscript.
